# Changes in Antioxidant Activity, Profile, and Content of Polyphenols and Tocopherols in Common Hazel Seed (*Corylus avellana* L.) Depending on Variety and Harvest Date

**DOI:** 10.3390/molecules25010043

**Published:** 2019-12-20

**Authors:** Karolina Pycia, Ireneusz Kapusta, Grażyna Jaworska

**Affiliations:** Department of Food Technology and Human Nutrition, Institute of Food Technology and Nutrition, College of Natural Sciences, University of Rzeszow, Zelwerowicza 4 St., 35-601 Rzeszow, Poland; ikapusta@univ.rzeszow.pl (I.K.); rrgjawor@cyf-kr.edu.pl (G.J.)

**Keywords:** hazelnuts, degree of ripeness, polyphenols

## Abstract

The aim of the study was to evaluate the influence of variety and the date of harvest of hazelnut seeds on their antioxidant potential and the profile and content of polyphenols and tocopherols. The research material included the hazelnut seeds of six varieties, harvested from July to September at equal 30-day intervals. Hazelnuts were analyzed for total fat content and antioxidant properties, whereby UPLC-PDA-ESI-MS analysis was used to determine the profile and content of polyphenols, and the HPLC method to determine the content of tocopherols. It was found that the content of fat and tocopherols in nuts increased with the ripening of the nuts. The highest fat content was found in walnut seeds of the Kataloński variety (September) and tocopherols in walnut seeds of the Olbrzym z Halle variety (177.67 mg/kg d.m.). In turn, antioxidant properties and total polyphenols content decreased with the later harvest date. The strongest antioxidant potential was found in the case of Cosford nuts harvested in July (66.93 mmol TE/100 g d.m.). These nuts were also characterized by the highest total polyphenol content (1704.9 mg/100 g d.m.). UPLC-MS analysis allowed the identification of 15 polyphenolic compounds such as phenolic acids, catechins and ellagic acid hexoside in nut seeds.

## 1. Introduction

Hazelnuts (*Corylus avellana* L.) are one of the most popular nuts in the world, valued for their pleasant taste and high nutritional value due to the content of valuable bioactive substances. The leading producer of hazelnuts is Turkey [1]. According to WHO, Turkey produced about 420,000 tons of hazelnuts in 2018, which accounts for 56% of world production [2]. Health claims made by the Food and Drug Administration (FDA) and the European Food Safety Authority (EFSA) indicate that the daily intake of nuts, including hazelnuts, should amount to 42.5 g (FDA)/30 g (EFSA) [3]. According to many researchers [4,5], frequent consumption of hazelnuts may contribute to reducing the risk of developing cardiovascular disease by reducing total cholesterol and low-density lipoprotein (LDL) cholesterol. These pro-health properties of hazelnuts are correlated with the presence of polyphenols in their chemical composition. Hazelnuts contain 291–875 mg/100 g polyphenols, which is a significant value, but among all nuts the most valuable source of these compounds are walnuts (1558–1625 mg/100 g) [1,6]. Among polyphenols present in hazelnuts there are phenolic acids, flavanols, flavon-3-ols, condensed tannins (procyanidins), and hydrolysable tannins. To a lesser extent there are also dihydrochalcones and flavonoles [7]. Condensed and hydrolysable tannins are the most abundant class of phenolic compounds in the chemical composition of walnuts and hazelnuts, which enhances their antioxidant potential [1]. Polyphenols or secondary plant metabolites can act synergistically with phytochemicals, thus reducing oxidative stress and the risk of inflammatory diseases [8]. Granata et al. [9] indicate that extracts of hazelnuts due to the high content of polyphenols have potent antioxidant properties not only, but also antimicrobial activities. However, the chemical composition of plant raw materials, including nuts, is not constant and changes during ripening or under the influence of biotic and abiotic factors. The biggest changes take place during maturation. Then the content of phenolic compounds changes dynamically. When fully ripe, edible parts of a walnuts fruit have a significantly lower level of polyphenols compared to unripe fruits [10]. However, the content of colored anthocyanins in the fruit skin or nut shell increases significantly. This trend is probably due to the defensive biological reactions of plants, as the tart and bitter taste characteristic of polyphenols repels herbivores [1]. During ripening, the chemical composition of the fruit changes in terms of quantity and quality, but the nature of these changes in the case of different hazelnut seed varieties is not fully known.

The aim of the study was to determine the changes in antioxidant properties, profile and content of polyphenols and tocopherols in hazelnut seeds differing in variety and harvest date (maturity stage).

## 2. Materials and Methods

### 2.1. Materials

Hazelnut seeds (*Corylus avellana* L.) of six varieties (Kataloński, Vebba Cenny, Barceloński, Lamberta, Cosford, Olbrzym z Halle) was used as a research material. The hazelnuts were harvested in 2017 from trees growing on plantations (‘Hanat’ 50°42′56″N 21°34′02″E, Poland). Hazelnuts were harvested during the growing season at regular intervals of 30 days (1 July, 1 August, and 1 September). The average laboratory test was 1 kg. After harvest, hazelnut samples were cleaned and shelled. The seeds were then partially crushed and dried in a freeze-dryer (−45 °C/24 h) (Alpha 1-2 LDplus, Martin Christ GmbH, Osterode am Harz, Germany). After drying, the test material was crushed and stored under freezing conditions (−18 °C) until analysis was performed.

### 2.2. Methods

#### 2.2.1. Analysis of Fat Content

The fat content of nuts was determined using the Soxhlet extractive weight method [11]. A sample of 5 g nuts was examined with 80 cm^3^ of petroleum ether. The extraction was performed in the Soxhlet’s apparatus. The extracted fat sample was dried at 105 °C. The analysis was performed in three repetitions.

#### 2.2.2. Analysis of Antioxidant Properties

##### 2.2.2.1. Extraction Process

Freeze-dried hazelnut seeds (1 g) were extracted with a solution of methanol (20 cm^3^) at a concentration of 70%. An ultrasound bath (Sonic 10, Polsonic, Poland) was used for extraction. The process was carried out for 35 min at the temperature of 25 ± 1 °C. After the extraction, the samples were centrifuged at 7000 rpm. The clear methanol extracts, after appropriate dilution, were used for the analysis of antioxidant properties and total polyphenols content.

##### 2.2.2.2. Antioxidant Properties and Total Polyphenols Content

The antioxidant activity of the analyzed hazelnut seeds was determined by the ABTS^+^ method [12], DPPH [13], and FRAP method [14]. In the case of the method using cation radical ABTS^+^ 0.03 cm^3^ diluted hazelnut seed extract, 3 cm^3^ of ABTS radical solution was applied. After a 6-minute reaction, a spectrophotometric measurement was made against distilled water at λ = 734 nm using the Hitachi UV-VIS spectrophotometer type U-2900 (Hitachi, Japan). For the DPPH (1,1-diphenyl-2-picrylhydrazyl) radical method, 2 cm^3^ of DPPH radical was added to 0.5 cm^3^ of methanol extract. Absorbance was measured after 10 min of reaction to methanol using a spectrophotometer at λ = 517 nm. The antioxidant activity of the color complex determined by FRAP was measured at λ = 593 nm. Spectrophotometric measurements were performed using the Nicolet Evolution 300 Type U-2900 UV-VIS spectrophotometer (HitachiThermo, Waltham, MA, USA). The antioxidant activities of the above-mentioned methods were calculated based on the standard curve. Antioxidant activity values were expressed in mmol TE (Trolox Equivalent)/100 g dry mass (d.m.). The determinations were made in three repetitions.

The total polyphenolic content (TPC) was determined by spectrophotometric method with Folin–Ciocalteu reagent [15]. Absorbance was measured at wavelength of 765 nm. Total polyphenols content was expressed in mg GAE/100 g d.m. The determinations were made in three repetitions.

#### 2.2.3. Analysis of Profile and Content of Polyphenols

The analysis of polyphenolic compounds profile was performed by ultra-efficient liquid chromatography coupled with a mass detector (UPLC-PDA-ESI-MS) (Waters, Micromass, Manchester, UK) [10,16,17]. The study used the Aquity liquid chromatograph (Waters, Micromass, Manchester, UK), coupled with a photodiode array (PDA) and a tandem quadrupole detector (TQD). The chromatographic distribution was carried out on C18 BEH column with dimensions of 100 mm × 2.1 mm and grain size of the order (1.7 µm) (Waters, Micromass, Manchester, UK). The eluent was a 0.1% aqueous solution of formic acid (eluent A) and a 40% aqueous solution of acetonitrile (eluent B). The following gradient was used in the analysis: 20% B and 80% A to 100% B and 0% A in 8 min. The total duration of the analysis was 9.5 min. Separation was performed at 50 °C at a moving phase velocity of 0.35 mL/min. The volume of the autosampler injection was 5 µL. The operating parameters of the mass sensor were as follows: Capillary voltage 3.5 kV, cone voltage 45 kV. The temperature of the ion source was 120 °C and the desolvation temperature 350 °C. Nitrogen with a flow rate of 800 L/h was used as a carrier gas. Polyphenols were detected in the negative ion mode in the range of *m*/*z* 120–2000. Mass-Lynx 4.1 (Waters) software was used to record and analyzed the data. The analyses were made in three repetitions. The determinations were made in two repetitions.

#### 2.2.4. Analysis of Tocopherols Content

Tocopherols (α, β, γ, δ) were analyzed by high-performance liquid chromatography (HPLC) using SYKAM chromatograph (Eresing, Germany) equipped with a fluorescence detector (Shimadzu RF 353) and 5C18-MS-II column (200 mm × 4.6 mm i.d., 5 µm Cosmosil) [10,17]. The column was developed in the isocratic system of methanol (100%) at a flow rate of 0.5 cm^3^/min. The column temperature was 50 °C and the injection volume 20 µL. The qualitative analysis was performed by comparing the retention times of the obtained peaks with the retention times of the standard substances. The quantitative analysis was carried out using the external standard method based on calibration curves made according to the dependence of the peak area on the concentration of the compound introduced into the column. Standard substances α, γ, and δ-tocopherols (Supelco, Bellefonte, PA, USA) were used for the analysis. A saponification of oil sample extruded from the tested nuts was analyzed. The analysis was made in three repetitions.

#### 2.2.5. Statistical Analysis

The obtained results were subjected to statistical analysis including two-way ANOVA. The Duncan test was used at a significance level of *p* < 0.01 to determine the significance of the differences between the mean values. LSD (least significant difference) was determined for average polyphenol content. Additionally, between the parameters characterizing the properties of the analyzed hazelnut seeds, the values of Pearson’s correlation coefficients were calculated, the significance of which was tested at the level of *p* < 0.01. Additionally, the principal component analysis (PCA) was performed. The statistical analysis was performed using Statistica 13.0 (StatSoft, Kraków, Poland).

## 3. Results and Discussion

### 3.1. Fat Content

**Figure 1:**
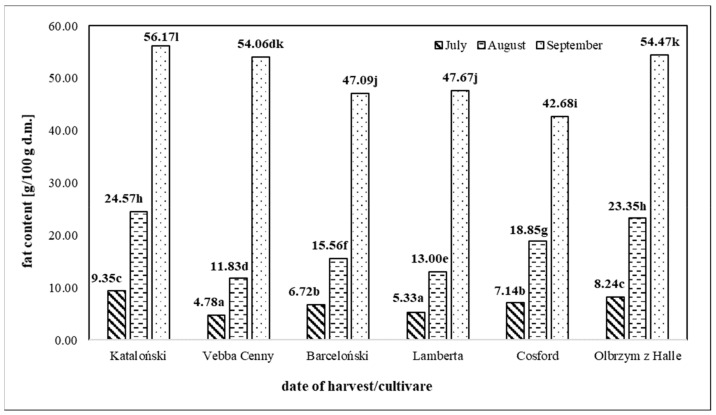
Average fat content of hazelnuts of different varieties, varying in degree of ripeness. Averages marked with the same letters do not differ significantly at the level (*p* < 0.05).

The fat content of nuts determines their energy and nutritional value. In soluble fats are compounds such as carotenoid dyes, tocopherols, and phytosterols, which are associated with the pro-health properties of nuts. Figure 1 shows the fat content in the seeds of the analyzed hazelnuts, differing in variety and harvest date, i.e., the degree of consumption maturity. In the case of nuts, fat is the predominant nutrient that determines their value and pro-health qualities. The two-way analysis of variance (ANOVA) confirmed a statistically significant (*p* < 0.001) effect of hazelnut variety, its phase of maturity, and the interaction between these factors on fat content. It was found that the mean value of this component in the examined seeds harvested in July, August, and September was 6.91 g/100 g d.m., 17.86 g/100 g d.m. and 59.43 g/100 g d.m. respectively. Therefore, the fat content of nut seeds increased with the ripening of the seeds. Among the nuts obtained for the study in September, the highest value of this parameter was found for the seeds of the Kataloński variety (56.17 g/100 g d.m.) and the lowest for the Cosford variety (42.68 g/100 g d.m.). Even so, the literature lacks information on the influence of the degree of maturity on the fat content of hazelnuts. Among data from Pycia et al. [10], a similar correlation was found between the fat content of walnut fruits and their harvest date. The quoted authors indicate that the content of this ingredient increased with the ripening and in walnuts harvested in September it was on average 22.64 g/100 g d.m. Data on the fat content of dry hazelnut seeds are quite numerous [18,19,20,21]. The average fat content of hazelnuts harvested in September, as determined in the study, was no less than the value of this parameter given in the literature for dry seeds. Savage et al. [18] analyzed six walnut varieties grown in New Zealand and found that the fat content ranged from 54.6% to 63.2%. Koyuncu [19] stated that the average fat content in seeds of Turkish hazelnuts was 60.94 g/100 g, and there was no statistical significance in the value of this parameter affected by the variety, which is not consistent with the observations presented in this study. In addition, the quoted authors found an increase in the fat content of nuts during storage, on average by 3.8 g/100 g over 12 months.

The average fat content in hazelnuts of 19 varieties grown in Portugal was 63.97% (59.20%–69.00%) [20]. Kornsteiner et al. [21] analyzed the fat content of nuts of various botanical origins and indicated that compared to others, hazelnuts have an average fat content of 60.2%. Moreover, the quoted authors indicate that the fat content is a species-specific characteristic of nuts and its content decreases in the following series: Macadamias > pecans > pines > Brazil nuts > walnuts > hazelnuts > almonds > pistachios > peanuts > cashews [21].

### 3.2. Antioxidant Potential with Total Polyphenol Content

Antioxidative potential determined by ABTS, DPPH and FRAP methods, as well as total polyphenols content in the analyzed hazelnut seeds differing in harvest date and cultivar were collected in Table 1. The results obtained clearly indicate that the strongest antioxidant properties and the related highest content of polyphenols in total were characteristic for unripe nuts obtained in July. Moreover, the numerical data indicated a statistically significant reduction in the value of the parameters in question, as nuts of all the varieties analyzed acquired maturity. This is confirmed by the conducted statistical analysis, which indicates a statistically significant effect of the harvest date, hazelnut variety and mutual interactions of these factors on the antioxidant capacity and total polyphenols content (*p* < 0.001). The DPPH radical method is widely used to assess the ability of antioxidants to capture free radicals. The absorption value at 517 nm decreases as the reaction between the antioxidant molecules and the DPPH radical progresses. Therefore, the faster the absorption decreases, the stronger the antioxidant activity of the extract in terms of its ability to supply the hydrogen atom [7,22]. The mean value of the antioxidative potential of nuts harvested in July was 27.38 mmol Trolox/100 g d.m., 56.86 mmol Trolox/100 g d.m. and 4.42 mmol Trolox/100 g d.m. respectively. In addition, the study showed that the highest value of antioxidant capacity was found in unripe Cosford nuts and the lowest value in Kataloński nuts. A similar correlation was observed in the remaining batches of samples. As the maturation progressed, a statistically significant decrease in antioxidant properties was observed, as the antioxidant potential of the nuts harvested in August was lower than that of those harvested in July by an average of 95% for ABTS, 86% for DPPH and 89% for FRAP. Nuts obtained in September, had the weakest antioxidant properties and the average value expressed using the ABTS method (as an exemplar) was 0.73 mmol Trolox/100g d.m. The statistical analysis showed a strong positive linear correlation between the parameters indicating antioxidant properties (ABTS and DPPH, FRAP) (r = 0.78, r = 0.76, *p* < 0.01, respectively). The parameters describing antioxidant properties were also strongly positively correlated with total polyphenols content (r = 0.95; *p* < 0.01). The total polyphenols content in the analyzed hazelnuts decreased with statistical significance, with the ripening of the nuts. The average value of this parameter for nuts harvested in July was about nine times higher than the average value for nuts harvested in September. Among the fruits harvested in July, the highest total polyphenol content was found in seeds from nuts of the Cosford variety (1704.29 mg/100 g d.m.). Information on the antioxidant potential and polyphenols in dry hazelnut seeds can be found in research [4,22,23,24], but there is little data on the effect of the variety and the date of harvest on these properties. Pelvan et al. [4] indicates that the total polyphenol content in seven commercial Turkish hazelnut varieties ranged from 6.21 to 14.31 mg/100 g d.m. and this value decreased as a result of the baking process. On the other hand, Marzocchi et al. [25] reported that the content of polyphenols in the dry hazelnut seeds of the Kataloński variety was 1245.27 mg/100 g d.m., and the value of this parameter increased with higher temperatures and longer baking times. Pycia et al. [10] analyzed the effect of the degree of maturity of walnuts on the content of polyphenols and antioxidant capacity. A similar tendency was demonstrated to see a decreased level of antioxidant properties with the acquisition of maturity. However, the presented research results do not coincide with some observations of other authors. Persic et al. [1] showed an increase in the concentration of polyphenols with the ripening of hazelnuts. The opposite tendency was observed in the case of the ripening of walnuts. A decrease in total polyphenols content during ripening is also observed in the case of fruits. Bizjak et al. [26] analyzing the relationship between the degree of maturity of apples on the content of metabolites, including polyphenols, found a decrease in the content of these substances in favor of an increased concentration of dyes in fruits such as anthocyanins.

The tart, astringent taste of unripe fruits is associated with the interaction of polyphenols with the protein of saliva rich in proline. The intensity of tart taste is a result of factors such as protein types (size, conformation structure, electrical charge), polyphenol types, protein and polyphenol concentrations, the presence of surfactants and the number and stereospecificity of the binding site on polyphenol and protein molecules [1]. However, at the time of consumption maturity, the astringent taste is already minimized, which is associated with a decrease in the concentration of polyphenols. Ballistreri et al. [27] investigated the effect of the degree of maturity on the content of total polyphenols, tocopherols, and polyphenolic profile in pistachios. The quoted authors have unequivocally demonstrated that the content of total polyphenols determined by the Folin–Ciocalteu method decreases with the maturation of pistachios, which is consistent with the presented research results. They also found that the content of polyphenols in immature pistachios was 185.52 mg GAE/100 g d.m.—higher than in mature and dry seeds by about 42%.

### 3.3. Profile and Content of Polyphenols

Table 2 shows the polyphenolic profile of hazelnuts of six varieties, differing in harvest date, and which were used for comparative analysis. As a result of UPLC-PDA-MS analysis, 15 polyphenolic compounds with strong antioxidant properties were identified in total.

Among the identified compounds, both free and esterified forms can be distinguished. The dominant group consisted of compounds classified as so-called tannins: both condensed (compounds **4** and **7**) and hydrolysable (compounds **3**, **5**, **6**, **8**, **9**, **11**, **12**, and **15**). The remaining compounds in the profile belonged to phenolic acids (compounds **1**, **2**, and **13**) and flavonoids (compounds **10** and **14**). The group of phenolic acids includes gallic acid, chlorogenic acid and caffeic acid derivative. The group of flavonols was formed by the quercetin hexosideand the kaempferd hexoside. Comparable profile of polyphenols in dry hazelnut seeds is given by Pelvan et al. [3]. The quoted authors identified gallic acid, protocatechuic acid, ferulic acid, sinapinic acid, and p-coumaric acid, whereby the first two dominated the phenolic acid spectrum in terms of content. The number of polyphenols in the seeds of three hazelnut varieties differing in the degree of ripeness (similar to that determined in the study), was identified by Persic et al. [1]. The authors quoted identified 12 polyphenolic compounds and 3 carboxylic acid derivatives as a result of UPLC-MS analysis of hazelnut seeds. Persic et al. [1] report that regardless of the stage of nut maturity, galloylquinic acid derivative I, caffeoyl hexoside, and catechin dominated the spectrum of polyphenols in terms of content. Pycia et al. [10]—in the profile of polyphenols of walnuts differing in the degree of ripeness and variety—identified as many as 26 compounds belonging mainly to the class of elagotanins. Therefore, the profile of polyphenols may depend on the botanical origin of the nuts. This was also confirmed by Ballistreri et al. [27]. The quoted authors, analyzing the spectrum of polyphenols of unripe and mature pistachio nuts, identified slightly different compounds in comparison to those presented in the paper, by Persic et al. [1] and Pycia et al. [10]. The authors mentioned above identified anthocyanins (cyanidin-3-galactoside and cyanidin-3-glucoside) and a large number of flavonoids (such as daidzein, genistein, daidzin, quercetin, eriodictyol, and genistin) in pistachio nut extract. It was shown that the content of all polyphenols decreased with the ripening of nuts.

The content of polyphenols analyzed by chromatography differed significantly both in relation to variety and harvest time (Table 3 and Table 4). Most polyphenols were found in fruits harvested the earliest, where the total content of analyzed compounds ranged from 512.17 to 9013.24 μg/100 g d.m. for Olbrzym z Halle and Cosford varieties respectively. The same factors also influenced the share of particular classes of polyphenols. Tannins constituted the dominant group of fruits harvested in July and their share in the total polyphenol content in the case of Barceloński variety was as high as 70%. In the remaining varieties collected in this period, the share of the other classes of identified compounds was relatively balanced, except for the Olbrzym z Halle variety, where phenolic acids were the dominant class. In subsequent harvest dates the total content of polyphenols decreased and the mutual ratio of the discussed classes of polyphenolic compounds changed. The share of tannins in the total polyphenols content decreased significantly and amounted to about 10%. Within this group, most of the identified compounds were present in trace amounts except for (+) catechin and (−) epicatechin gallate. The remaining polyphenols groups in fruits harvested in the following dates were at a comparable level, with a significant increase in the share of two flavonole derivatives. The study observed fluctuations in the level of phenolic acids, because the content of gallic acid and chlorogenic acid in the seeds harvested in August and September was respectively higher and lower than those obtained in July. The decrease in polyphenols as the hazelnuts mature is consistent with the trend observed for walnuts [10] and pistachios [3]. Persic et al. [1] observed in their studies an increase in hydroxybenzoic acid, hydroxycinnamic acid and flavanol content as hazelnuts ripened. However, the same researchers have confirmed that the changes in the profile and polyphenol content of nut seeds during ripening depend on their botanical origin. Persic et al. [1] in their study of walnuts found a decrease in the total polyphenol content during the ripening process. The quoted authors clearly indicate that hazelnuts contain much less polyphenols than walnuts. Similar observations had already been made by Bolling et al. [6] and Kalogeropoulos et al. [28]. Pycia et al. [10,17], when analyzing dry walnuts in terms of total polyphenols content and changes in this parameter depending on the maturity, found that they give higher average polyphenol content than determined in the study. The consumption of hazelnuts characterized by a high level of polyphenols and a favorable profile of these compounds can contribute to health improvement, because Machado de Souza et al. [29] indicate that nuts with high antioxidant potential are helpful in the treatment and prevention of cardiovascular diseases, cancer and reduction of oxidative stress.

### 3.4. Profile and Content of Tocopherols

Tocopherols are the basic biological-active substances found in vegetable oils. They are a biological form of vitamin E, often referred to as the vitamin of youth. The analyzed hazelnuts differed in terms of tocopherol content and the degree of maturity and variety were statistically significant factors influencing this parameter. The statistical analysis illustrated that the average tocopherol content in the analyzed hazelnuts was influenced by the varieties, harvest time and interactions between the factors mentioned above (*p* < 0.001). The value of this parameter increased with the ripeness, because the average total tocopherol content in nuts harvested in July, August, and September was 3.53 mg/kg d.m., 9.08 mg/kg d.m., and 144.86 mg/kg d.m. respectively (Table 5). This is in line with the observations of Pycia et al. [10] who found that the tocopherol content of walnut fat increased as they mature. Ballistreri et al. [27] reported that the content of tocopherols in mature pistachios (13.5 mg/100 g d.m.) was lower than in immature pistachios (19.9 mg/100 g d.m.). Nuts of the different varieties also differed in terms of the value of the parameter in question. The highest number of tocopherols among nuts harvested in July and August was found in nuts of the Kataloński variety, and nuts of the Olbrzym z Halle variety had the highest number of tocopherols in comparison to other nuts harvested at that time (177.67 mg/kg d.m.). The analysis of the tocopherol spectrum showed that the dominant fraction was α-tocopherol, whose average share in the nut oil obtained in July, August, and September was 68%, 52%, and 67%, respectively. Moreover, the content of the α-tocopherol fraction correlated positively with the content of delta tocopherols and the sum of tocopherols (r = 0.62, r = 0.95, *p* < 0.01, respectively). Moreover, individual tocopherol fractions (γ, δ, α) and their sum of contents strongly positively correlated with fat content (r = 0.54, r = 0.87, r = 0.83, r = 0.94, *p* < 0.01, respectively). The literature lacks data on the effect of the degree of ripeness of nuts on tocopherols. The authors analyzed the chemical composition of dry hazelnut seeds of various varieties.

Marzocchi et al. [25] analyzed the profile and content of tocopherols in oil from Kataloński hazelnuts from Polish plantations and found higher content of these substances in comparison to those presented in the study (81.15 mg/100 g). Such a significant difference may result from different cultivation conditions, analysis procedures or degree of ripeness. This is since, according to the authors quoted above, the nuts examined by them were fully ripe. Durmaz and Gokmen [2] indicated that the tocopherols content of hazelnut oil ranged from 479.44 mg/kg to 512.75 mg/kg. The study showed that the leading tocopherols fraction in hazelnut oil is α-tocopherol, with an average percentage in the nut oil obtained in September of 67%. The demonstrated dominance of this fraction in the tocopherols profile is consistent with the observations of other authors [2,25,30]. Alasalvar et al. [30] and Durmaz and Gokmen [2] reported slightly higher values of 88% and 80% respectively. From the literature data it can be concluded that the dominance of individual fractions in the tocopherol spectrum in nut fat is a species characteristic. Ballistreri et al. [27] analyzed the profile of tocopherols in pistachio nut fat differing in maturity and indicated the dominance of γ-tocopherol. Meanwhile, Pycia et al. [17] studied the content and profile of tocopherols in walnuts and indicated that the domination of particular forms of tocopherols in fat depended on the degree of ripeness and variety of nuts. Significant tocopherol losses are recorded as a result of the refining process as, according to Alasalvar et al. [30] and Durmaz and Gokmen [2], the refining process to which the oil was subjected after pressing hazelnut seed results in a significant reduction in the concentration of tocopherols and polyphenols. Furthermore, it results in a complete loss of the leading carotenoids in the crude oil, i.e., lutein and zeaxanthin. Significant loss of biologically active compounds due to overlapping of particular refining stages is also connected with a reduction in the antioxidative capacity of such oil. This proves that the tocopherol content of hazelnut oil is a function not only of the degree of ripeness of the variety, but also of a number of technological processes. Tocopherols are valuable health-promoting substances. The participation of tocopherols in the diet reduces LDL cholesterol, reduces the risk of atherosclerosis and promotes the fight against obesity Machado de Souza et al. [29].

### 3.5. Principal Component Analysis

**Figure 2:**
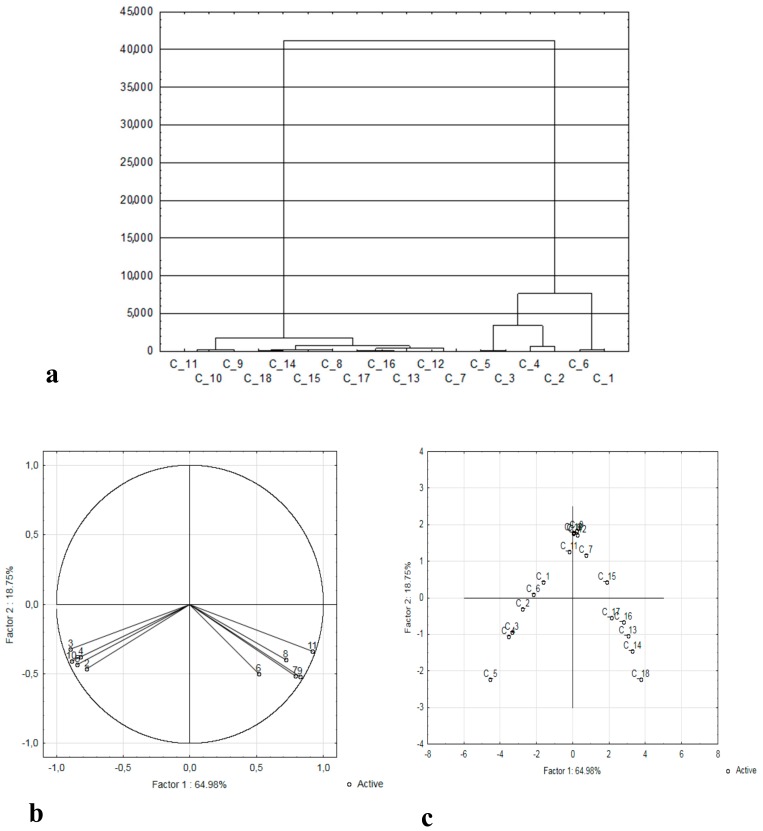
Analysis of the main components: (**a**) dendrogram, (**b**) distribution of analyzed parameters (1-ABTS, 2-DPPH, 3-FRAP, 4-TPC, 5-γ-toc, 6-δ-toc, 7-α-toc, 8-total toc., 9-phenols content, 10-fat content); (**c**) distribution of hazelnut samples. (Kataloński, Vebba Cenny, Barceloński, Lamberta, Cosford, Olbrzym z Halle collected in July C1–C6, August C7–C12, September C13–C18).

The analytical data obtained was statistically processed using cluster analysis and principal components analysis (PCA). As a result of the Ward method grouping, a dendrogram was obtained (Figure 2a). The analysis of the obtained clusters allowed the distinguishing of three separate groups of analyzed hazelnut samples. The first focused on those with the highest antioxidant potential (samples C1–C6) and the second focused on the remainder. The second group of samples can be divided into two categories. One of them is the sample of nuts with the lowest antioxidant potential and the highest tocopherols content.

The relationships between input variables and the main components are shown in Figure 2b. This projection shows the distribution of characteristics of particular analyzed parameters on the plane formed by two selected factors. PCA explains 83.73% of total variability, with the first and second factors explaining 64.98% and 18.75% of intergroup variances respectively. When analyzing the presented distribution of variables, they can be divided into two categories. The first one focuses on parameters related to antioxidant properties (ABTS, FRAP, DPPH, ABTS, TPC, and the quantity of polyphenols determined by UPLC-MS). The second category consists of parameters related to the content of tocopherols and fat. The arrangement of both parameter groups in close proximity to the circle indicates that a large part of the information contained in the input variable is carried by the main components. The arrangement of variables from the first and second category in relation to each other indicates their mutual strong positive correlation. This is confirmed by Pearson’s correlation values for parameters showing antioxidant properties, tocopherols and fat content.

Figure 2c shows the projection of samples of various hazelnut varieties with different degrees of maturity on the plane of the factors. The analysis of the relationship between factor 1 and factor 2 shows significant similarities between nuts harvested on particular dates (July, August, September). Close proximity to each other confirms their similarity in terms of chemical composition.

## 4. Conclusions

The results of the study unequivocally show that the antioxidant properties and related pro-health properties of nuts depends significantly on the time at which they were harvested, i.e., their maturity phase. This factor also determines the fat content, which increases with the ripening of the hazelnuts. The average fat content of nuts harvested in September was 59.43 g/100 g d.m. A similar tendency was also observed in the case of tocopherols. It should be noted that these parameters were strongly positively correlated with each other. Moreover, the tocopherol spectrum was dominated by the α-tocopherols fraction, which is the most valuable form of vitamin E. The study also showed that antioxidant properties and polyphenols content decreased with the ripening of nuts. The strongest antioxidant potential was found in nuts harvested in July. In polyphenols profiles 15 compounds with strong antioxidant properties were identified. Cosford nuts harvested in July were characterized by the highest content of polyphenols (9013.24 µg/100 g d.m.). This shows that unripe hazelnuts are a valuable natural source of antioxidants. Their extracts can be used to compose functional food with high antioxidant capacity.

## Figures and Tables

**Table 1:** Antioxidative potential of hazelnuts of various cultivars, in various stages of ripeness.

Cultivar	ABTS	DPPH	FRAP	Total Polyphenols
	[mmol Trolox/100g d.m.]			[mg/100g d.m.]
	July			
**Kataloński**	2.14 ^c^ ± 0.83	46.71 ^h^ ± 1.27	2.06 ^f^ ± 0.13	438.6 ^h^ ± 1.11
**Vebba Cenny**	18.60 ^e^ ± 1.91	58.82 ^k^ ± 1.91	3.64 ^g^ ± 0.17	585.6 ^i^ ± 2.39
**Barceloński**	14.74 ^d^ ± 2.19	56.58 ^j^ ± 1.34	6.16 ^j^ ± 0.11	1007.0 ^k^ ± 4.79
**Lamberta**	27.76 ^g^ ± 1.36	60.13 ^k^ ± 3.52	3.84 ^i^ ± 0.19	877.1 ^j^ ± 0.64
**Cosford**	66.93 ^h^ ± 3.34	65.91 ^l^ ± 1.02	7.52 ^k^ ± 0.17	1704.9 ^l^ ± 2.29
**Olbrzym z Halle**	24.12 ^f^ ± 0.65	53.04 ^i^ ± 1.05	3.29 ^g^ ± 0.06	462.3 ^h^ ± 0.66
	**August**			
**Kataloński**	0.83 ^a^ ± 0.03	5.42 ^de^ ± 0.15	0.26 ^bc^ ± 0.02	196.0 ^f^ ± 0.70
**Vebba Cenny**	1.44 ^a^ ± 0.08	7.71 ^f^ ± 0.29	0.47 ^d^ ± 0.03	195.3 ^f^ ± 0.27
**Barceloński**	0.23 ^a^ ± 0.18	4.72 ^cd^ ± 0.17	0.19 ^ab^ ± 0.01	103.6 ^bcd^ ± 0.15
**Lamberta**	0.73 ^a^ ± 0.11	7.56 ^f^ ± 0.16	0.41 ^cd^ ± 0.02	121.7 ^de^ ± 0.32
**Cosford**	4.14 ^b^ ± 0.13	11.42 ^g^ ± 0.14	1.07 ^e^ ± 0.04	385.9 ^g^ ± 1.11
**Olbrzym z Halle**	0.65 ^a^ ± 0.12	7.12 ^ef^ ± 0.13	0.39 ^cd^ ± 0.00	140.4 ^e^ ± 0.15
	**September**			
**Kataloński**	0.47 ^a^ ± 0.13	5.45 ^de^ ± 0.17	0.30 ^bc^ ± 0.01	91.6 ^abc^ ± 0.18
**Vebba Cenny**	0.42 ^a^ ± 0.15	0.36 ^a^ ± 0.01	0.08 ^a^ ± 0.01	68.5 ^a^ ± 0.20
**Barceloński**	0.55 ^a^ ± 0.07	2.96 ^bc^ ± 0.02	0.15 ^ab^ ± 0.00	85.9 ^abc^ ± 0.44
**Lamberta**	0.69 ^a^ ± 0.10	0.71 ^a^ ± 0.04	0.18 ^ab^ ± 0.01	112.5 ^cd^ ± 0.48
**Cosford**	1.83 ^a^ ± 0.06	10.37 ^g^ ± 0.05	1.00 ^e^ ± 0.05	141.4 ^e^ ± 0.38
**Olbrzym z Halle**	0.43 ^a^ ± 0.06	2.04 ^ab^ ± 0.27	0.30 ^bc^ ± 0.01	86.1 ^ab^ ± 0.03
	**Two-Factor ANOVA-*p***			
**Factor 1**	<0.001	<0.001	<0.001	<0.001
**Factor 2**	<0.001	<0.001	<0.001	<0.001
**Factor 1 × Factor 2**	<0.001	<0.001	<0.001	<0.001

Mean values of three replicates marked with the same letter in the column do not differ significantly at a significance level of 0.05. Factor 1—harvest time (degree of nut ripeness). Factor 2—cultivar of nuts. Factor 1 × Factor 2—interaction between harvest time and the cultivar of nuts. ± standard deviation.

**Table 2:** Individual phenolic compounds identified by UPLC-PDA-MS/MS.

Compounds		Rt	[M − H] *m*/*z*	
		min	MS	MS/MS
**1**	Gallic acid	1.10	169	125
**2**	Chlorogenic acid	1.91	353	191
**3**	Digalloyl ester of procyanidin dimer	2.36	881	577.289
**4**	(+) catechin	2.89	289	137
**5**	Digalloyl ester of procyanidin dimer	3.34	881	577.289
**6**	Ellagic acid hexoside	3.81	463	301
**7**	Procyanidin dimer	4.05	577	289
**8**	Ellagic acid hexoside	4.16	463	301
**9**	Ellagic acid hexoside	4.24	463	301
**10**	Quercetin hexoside	4.62	463	301
**11**	(−) epicatechin gallate	4.72	441	331.289
**12**	Valenoic acid dilactone	5.13	469	451.425
**13**	Unspecified derivative of caffeic acid	5.33	563	179
**14**	Kaempferol hexoside	5.40	447	285
**15**	Ellagic acid pentoside	6.05	433	301

**Table 3:** Quantitative composition of polyphenols identified in hazelnuts harvested in July and August (µg/100 g d.m.).

Compound	Rt	[M − H] *m*/*z*	Cultivar											
	min	MS	Kataloński	Vebba Cenny	Barceloński	Lamberta	Cosford	Olbrzym z Halle	Kataloński	Vebba Cenny	Barceloński	Lamberta	Cosford	Olbrzym z Halle
			July						August					
**1**	1.10	169	85.80 ± 9.14	24.77 ± 2.88	nd	84.46 ± 2.23	76.56 ± 3.27	nd	159.43 ± 2.78	nd	nd	213.20 ± 4.12	118.55 ± 1.97	nd
**2**	1.91	353	nd	109.85 ± 6.07	nd	257.23 ± 8.21	154.91 ± 5.16	17.99 ± 1.08	23.58 ± 1.18	42.78 ± 1.81	29.37 ± 1.40	49.88 ± 2.35	30.37 ± 1.63	67.12 ± 3.54
**3**	2.36	881	nd	177.32 ± 5.55	441.39 ± 6.23	nd	275.09 ± 4.87	21.601.25	20.90 ± 1.12	10.04 ± 0.73	nd	19.58 ± 1.28	nd	25.90 ± 1.63
**4**	2.89	289	136.45 ± 11.40	585.05 ± 19.10	5806.03 ± 88.20	398.87 ± 7.78	1729.41 ± 35.50	29.11 ± 1.33	62.88 ± 2.04	10.99 ± 0.81	67.47 ± 2.12	nd	nd	46.09 ± 2.78
**5**	3.34	881	nd	nd	nd	nd	64.61 ± 2.40	nd	nd	nd	nd	nd	nd	nd
**6**	3.81	463	124.90 ± 6.89	100.89 ± 4.95	nd	195.26 ± 2.55	156.90 ± 4.76	29.45 ± 2.11	nd	nd	nd	nd	nd	nd
**7**	4.05	577	152.42 ± 8.28	141.95 ± 5.93	nd	147.21 ± 2.26	141.66 ± 3.72	nd	nd	nd	nd	nd	nd	nd
**8**	4.16	463	83.19 ± 2.28	nd	nd	83.41 ± 0.45	118.55 ± 4.35	nd	nd	nd	nd	nd	dd	nd
**9**	4.24	463	129.17 ± 5.16	124.52 ± 3.12	nd	nd	183.16 ± 5.55	nd	nd	nd	nd	nd	nd	nd
**10**	4.62	463	1629.91 ± 28.30	2709.40 ± 41.26	nd	2050.73 ± 77.80	2528.15 ± 46.70	nd	nd	nd	nd	52.07 ± 1.22	99.84 ± 2.61	nd
**11**	4.72	441	562.25 ± 11.40	489.42 ± 10.04	115.18 ± 2.03	409.47 ± 8.60	609.78 ± 6.24	nd	121.98 ± 5.46	105.64 ± 1.91	nd	94.33 ± 3.30	nd	103.06 ± 4.35
**12**	5.13	469	nd	489.02 ± 9.06	114.002.98	nd	164.85 ± 3.49	nd	nd	105.00 ± 2.07	nd	nd	nd	nd
**13**	5.33	563	187.60 ± 6.35	235.20 ± 9.72	nd	nd	169.06 ± 3.35	nd	nd	143.21 ± 3.26	nd	nd	nd	nd
**14**	5.40	447	1149.27 ± 58.70	1876.80 ± 54.30	777.91 ± 12.30	2371.45 ± 4.29	1710.09 ± 30.40	304.97 ± 7.12	135.94 ± 3.06	nd	31.58 ± 2.08	483.46 ± 5.55	225.52 ± 4.69	148.19 ± 5.67
**15**	6.05	433	426.53 ± 12.40	502.15 ± 6.96	406.17 ± 5.67	719.05 ± 7.52	930.45 ± 22.01	9.05 ± 0.85	25.38 ± 1.49	nd	13.43 ± 1.15	56.06 ± 3.34	29.24 ± 1.04	nd
**TOTAL**			**4667.48**	**7366.00**	**8893.08**	**6717.13**	**9013.24**	**512.17**	**550.09**	**312.01**	**141.85**	**968.58**	**503.53**	**390.35**
**LSD**			**14.57**	**13.76**	**11.67**	**12.16**	**12.11**	**2.29**	**2.44**	**1.76**	**1.68**	**3.02**	**2.38**	**3.59**

LSD—least significant difference. ±—standard deviation. nd—not detected.

**Table 4:** Quantitative composition of polyphenols identified in hazelnuts harvested in September (µg/100 g d.m.).

Compound	Rt	[M − H] *m*/*z*	Cultivar					
	min	MS	Kataloński	Vebba Cenny	Barceloński	Lamberta	Cosford	Olbrzym z Halle
			September					
**1**	1.10	169	28.29 ± 1.12	nd	95.92 ± 5.38	36.40 ± 1.28	nd	nd
**2**	1.91	353	33.63 ± 2.33	41.70 ± 3.54	106.53 ± 6.07	19.61 ± 0.99	38.73 ± 2.01	32.91 ± 2.22
**3**	2.36	881	nd	nd	nd	nd	nd	nd
**4**	2.89	289	21.57 ± 1.98	53.72 ± 4.12	91.64 ± 4.89	37.12 ± 2.12	nd	59.04 ± 1.55
**5**	3.34	881	nd	nd	nd	nd	nd	nd
**6**	3.81	463	nd	nd	nd	nd	nd	nd
**7**	4.05	577	nd	nd	nd	nd	nd	nd
**8**	4.16	463	nd	nd	nd	nd	nd	nd
**9**	4.24	463	nd	nd	nd	nd	nd	nd
**10**	4.62	463	150.12 ± 4.91	nd	nd	97.44 ± 3.87	138.67 ± 3.22	79.10 ± 3.03
**11**	4.72	441	nd	nd	nd	nd	nd	nd
**12**	5.13	469	nd	nd	nd	nd	nd	nd
**13**	5.33	563	nd	nd	nd	nd	nd	nd
**14**	5.40	447	311.56 ± 7.78	234.80 ± 6.27	372.31 ± 7.11	247.66 ± 5.05	360.02 ± 6.92	292.78 ± 6.63
**15**	6.05	433	nd	nd	37.49 ± 2.98	31.85 ± 0.96	nd	nd
**TOTAL**			**545.17**	**330.22**	**803.42**	**470.07**	**537.42**	**463.84**
**LSD**			**3.62**	**4.64**	**5.88**	**2.37**	**4.05**	**3.35**

LSD—least significant difference. ±—standard deviation. nd—not detected.

**Table 5:** Content of tocopherols in hazelnuts of different cultivars, in different stages of ripeness.

Cultivar	α-Tocopherol		γ-Tocopherol		Sum β and δ-Tocopherol		Total
	mg/kg d.m.	%	mg/kg d.m.	%	mg/kg d.m.	%	mg/kg d.m.
	**July**						
**Kataloński**	5.50 ^bc^ ± 0.16	78.6	0.50 ^cd^ ± 0.17	7.1	1.00 ^a^ ± 0.00	14.3	7.00 ^ab^ ± 0.31
**Vebba Cenny**	1.17 ^a^ ± 0.17	46.7	0.17 ^ab^ ± 0.08	6.6	1.33 ^a^ ± 0.00	53.3	2.50 ^a^ ± 0.17
**Barceloński**	2.57 ^ab^ ± 0.10	60.6	0.50 ^cd^ ± 0.16	11.8	1.17 ^a^ ± 0.11	27.6	4.23 ^a^ ± 0.30
**Lamberta**	1.33 ^a^ ± 0.32	70.0	0.00 ^a^ ± 0.00	0.0	0.83 ^a^ ± 0.14	30.0	2.67 ^a^ ± 0.33
**Cosford**	2.33 ^ab^ ± 0.21	72.0	0.00 ^a^ ± 0.00	0.0	0.83 ^a^ ± 0.11	28.0	3.16 ^a^ ± 0.13
**Olbrzym z Halle**	1.33 ^a^ ± 0.01	80.0	0.00 ^a^ ± 0.00	0.0	0.33 ^a^ ± 0.01	20.0	1.67 ^a^ ± 0.01
	**August**						
**Kataloński**	4.83 ^abc^ ± 1.15	19.7	0.33 ^bc^ ± 0.03	1.3	20.00 ^c^ ± 0.33	79.0	25.33 ^c^ ± 1.33
**Vebba Cenny**	2.00 ^ab^ ± 0.00	39.6	0.83^e^ ± 0.07	16.5	2.27 ^a^ ± 0.27	44.9	5.05 ^ab^ ± 0.13
**Barceloński**	3.67 ^ab^ ± 0.01	68.7	0.50 ^cd^ ± 0.07	9.4	1.17 ^a^ ± 0.12	21.9	5.33 ^ab^ ± 0.50
**Lamberta**	2.50 ^ab^ ± 0.07	51.7	1.33 ^f^ ± 0.01	27.6	1.00 ^a^ ± 0.00	20.7	4.83 ^ab^ ± 0.17
**Cosford**	7.50 ^c^ ± 0.50	72.6	0.67 ^de^ ± 0.02	6.4	2.17 ^a^ ± 0.17	21	10.33 ^b^ ± 0.83
**Olbrzym z Halle**	2.50 ^ab^ ± 0.18	63.6	0.00 ^a^ ± 0.00	0.0	1.43 ^a^ ± 0.36	36.4	3.93 ^a^ ± 0.35
	**September**						
**Kataloński**	129.33 ^g^ ± 1.00	77.2	1.50 ^g^ ± 0.17	0.9	36.67 ± 5.67	21.9	167.50 ^g^ ± 6.50
**Vebba Cenny**	37.00 ^d^ ± 0.05	28.5	36.33 ^h^ ± 0.33	27.2	57.33 ^f^ ± 0.67	37.0	129.67 ^e^ ± 1.00
**Barceloński**	72.67 ^e^ ± 7.01	83.8	0.00 ^a^ ± 0.00	0.0	14.00 ^b^ ± 0.31	16.1	86.67 ^d^ ± 7.33
**Lamberta**	133.67 ^h^ ± 2.67	82.5	0.00 ^a^ ± 0.00	0.0	28.33 ^d^ ± 4.67	17.5	162.00 ^g^ ± 6.62
**Cosford**	126.33 ^g^ ± 4.67	86.7	2.83 ^h^ ± 0.17	1.9	16.50 ^b^ ± 0.83	11.3	145.67 ^f^ ± 5.83
**Olbrzym z Halle**	77.67 ^f^ ± 0.01	43.7	53.00 ^i^ ± 0.31	29.8	47.00 ^e^ ± 0.30	26.4	177.67 ^h^ ± 0.83
	**Two-Factor ANOVA-*p***						
**Factor 1**	<0.001		<0.001		<0.001		<0.001
**Factor 2**	<0.001		<0.001		<0.001		<0.001
**Factor 1 × Factor 2**	<0.001		<0.001		<0.001		<0.001

Mean values of three replicates marked with the same letter in the column do not differ significantly at the significance level of 0.05. Factor 1—harvest time (degree of nut ripeness). Factor 2—cultivar of nuts. Factor 1 × Factor 2—interactions between the harvest time nuts and the cultivar of nuts. ± standard deviation.
